# A comparison of reinforcing effectiveness and drug-seeking reinstatement of 2-fluorodeschloroketamine and ketamine in self-administered rats

**DOI:** 10.3389/fnmol.2022.972798

**Published:** 2022-09-12

**Authors:** Han Du, Miaojun Lai, Dingding Zhuang, Dan Fu, Yiying Zhou, Shanshan Chen, Fangmin Wang, Zemin Xu, Huifen Liu, Youmei Wang, Peng Xu, Wenhua Zhou

**Affiliations:** ^1^Zhejiang Provincial Key Laboratory of Addiction Research, Ningbo Kangning Hospital, School of Medicine, Ningbo University, Ningbo, China; ^2^Key Laboratory of Drug Monitoring and Control, Drug Intelligence and Forensic Center, Ministry of Public Security, Beijing, China

**Keywords:** new psychoactive substance, PCP, ketamine, BDNF, GSK-3β

## Abstract

2-Fluorodeschloroketamine (2F-DCK), a structural analog of ketamine, has been reported to cause impaired consciousness, agitation, and hallucination in abuse cases. It has similar reinforcing and discriminative effects as ketamine. However, the reinforcing efficacy and drug-seeking reinstatement of this analog have not been clarified to date. In this study, the effectiveness of 2F-DCK and ketamine was compared using a behavioral economics demand curve. The reinstatement of 2F-DCK- and ketamine-seeking behaviors induced by either conditioned cues or self-priming was also analyzed. Rats were intravenously self-administered 2F-DCK and ketamine at a dose of 0.5 mg/kg/infusion under a reinforcing schedule of fixed ratio 1 (FR1) with 4 h of daily training for at least 10 consecutive days. The elasticity coefficient parameter α and the essential value of the demand curve in the two groups were similar. Both groups of rats showed significant drug-seeking behavior induced either by conditional cues or by 2F-DCK and ketamine priming. Moreover, the α parameter was inversely related to the degree of reinstatement induced by cues or drug priming in both groups. In total, the expression levels of brain-derived neurotrophic factor (BDNF) and phosphorylated cAMP response element-binding protein (p-CREB) in the nucleus accumbens in both extinguished and reinstated rats were significantly lower than those in the control. The expression of total Akt, glycogen synthase kinase (GSK)-3β, mammalian target of rapamycin (mTOR), and extracellular signal-related kinase (ERK) also decreased, but p-Akt, p-GSK-3β, p-mTOR, and p-ERK levels increased in both extinguished and reinstated rats. This is the first study to demonstrate that 2F-DCK has similar reinforcing efficacy, effectiveness, and post-withdrawal cravings as ketamine after repeated use. These data suggest that the downregulation of CREB/BDNF and the upregulation of the Akt/mTOR/GSK-3β signaling pathway in the nucleus accumbens may be involved in ketamine or 2F-DCK relapse.

## Introduction

Ketamine, a dissociative anesthetic and psychedelic compound, has rapid-acting antidepressant activity in patients with major depression (Berman et al., 2000) and resistant depression (Zarate et al., 2006). Ketamine's efficacy, however, is transient, and repeated clinical use often leads to drug dependence or use disorders (Krystal et al., 1994). Ketamine is known for its abuse liability, which poses a major challenge for its clinical use in depression and other mental disorders (Liu et al., 2016). Preclinical studies have shown that ketamine produces discriminative (Chiamulera et al., 2016) and reinforcing effects in women and men (Wright et al., 2017). 2-Fluorodeschloroketamine (2F-DCK) is a novel psychoactive ketamine derivative that has been detected in wastewater treatment plants (Shao et al., 2021). Sporadic clinical reports have described the use of 2F-DCK to induce a dissociated state (Domanski et al., 2021) and identified the 2F-DCK concentration in the illegal range in forensic blood and hair samples of drivers under the influence of drugs (Davidsen et al., 2020). The clinical effects in patients exposed to 2F-DCK are predominantly impaired consciousness, agitation, abnormal behavior, hypertension, and tachycardia (Tang et al., 2020). 2F-DCK has recently emerged as a substitute for ketamine in drug abusers. However, 2F-DCK has not been controlled or regulated in many countries, which may be partly related to the lack of evidence regarding its abuse potential.

The reinforcing effects of drugs are fundamental to the development of drug addiction and are intrinsic to most rodent models of addiction-like behaviors (Koffarnus et al., 2012). 2F-DCK was recently shown to produce self-administration, generalized to ketamine discriminative stimuli and induce conditioned place preference in rats (Li et al., 2022). Self-administration of ketamine or 2F-DCK determines their functions as reinforcers but does not provide quantitative information about their reinforcing effectiveness (Huskinson et al., 2014). Behavioral economics approaches permit the quantification of reinforcing effects, placing a relative value on different drug reinforcers (Galuska et al., 2011; Koffarnus et al., 2012). Behavioral economics curves are independent of the magnitude (dose) of the reinforcer, allowing each reinforcing stimulus to be assigned a single number that reflects its reinforcing effectiveness (Hursh and Silberberg, 2008). Addicted individuals are highly susceptible to relapse when exposed to the self-administered drug and drug-associated cues even after extensive periods of abstinence (Kuijer et al., 2020). Reinstatement procedures in animal models are thought to resemble drug-seeking and relapse in humans (Epstein and Preston, 2003; Shaham et al., 2003). Preclinical data have shown reinstatement of ketamine-seeking behavior induced by cues and ketamine priming after ketamine self-administration (Huang et al., 2015). However, to date, the reinforcing effectiveness and reinstatement of drug seeking by 2F-DCK are still unclear.

Ketamine is proposed to selectively block *N*-methyl-d-aspartate (NMDA) receptors expressed on GABAergic inhibitory interneurons, which results in the disinhibition of pyramidal neurons, enhancement of brain-derived neurotrophic factor (BDNF) release, and subsequent promotion of protein synthesis (Zanos and Gould, 2018). The antidepressant effects of ketamine are mediated, at least in part, by molecular adaptations, resulting in long-lasting synaptic changes in the mesolimbic brain regions known to regulate natural and drug rewards (Zanos and Gould, 2018). Silencing of glycogen synthase kinase (GSK)-3β in the nucleus accumbens (NAc) increases depression- and addiction-related behavior (Crofton et al., 2017). Ketamine at a lower dose induces behavioral sensitization, which is accompanied by an increase in the spine density in the NAc and changes in protein expression in pathways commonly implicated in addiction (Strong et al., 2017). Three weeks of abstinence from ketamine was associated with increased dendritic mushroom spines in the NAc (Strong et al., 2019). The mammalian target of rapamycin (mTOR) inhibitor rapamycin blocks the effects of ketamine (Sabino et al., 2013). However, much remains to be known about the long-term effects of ketamine and the neurobiological mechanisms underlying its addiction (Kokane et al., 2020; Sial et al., 2020).

In the present study, we used a behavioral economics approach to compare the reinforcing effectiveness of 2F-DCK and ketamine, and compared the reinstatement of 2F-DCK- and ketamine-seeking behaviors induced by either conditioned cues or themselves. The expression of BDNF, CREB, extracellular signal-related kinase (ERK), Akt, GSK-3β, mTOR, and their phosphorylation in the NAc after extinction or reinstatement induced by both cues and priming were measured by Western blotting. These studies will provide evidence for the difference in abuse potential between 2F-DCK and ketamine and the molecular mechanisms underlying the relapse of 2F-DCK and ketamine in rats.

## Materials and methods

### Subjects

Male Sprague–Dawley rats (280–300 g) purchased from the Experimental Animal Center of Zhejiang Province, China, were housed in a temperature- and humidity-controlled ventilated colony room with a reversed 12-h light/dark cycle (lights onset 20:00 h, offset 8:00 h). The temperature was maintained at 22–24°C, and humidity levels were stable (50–70%). The experimental sessions were performed during the dark period. Food and water were provided *ad libitum* in the home cage. All experiments were conducted in accordance with the Eighth Edition of the Guide for Care and Use of Laboratory Animals (I.f.L.A. Research., 2011). This study was approved by the Hospital Ethics Committee for Animal Use.

Ketamine crystalline powder was provided by the Drug Intelligence and Forensic Center of the Ministry of Public Security, China. The crystalline 2F-DCK powder was provided by the Drug Laboratory of the Narcotic Control Division of the Nanjing Public Security Bureau, China. All the drugs were dissolved in physiological saline.

### Surgery

After acclimation to the environment for 7 days, the animals were anesthetized with sodium pentobarbital (50 mg/kg, i.p.). Each rat was subsequently implanted with a chronically indwelling intravenous catheter (Silastic; length, 3.5 cm; inner diameter, 0.5 mm; outer diameter, 0.94 mm) into the right external jugular vein, and the other end of the catheter (10 cm, PE20) was passed subcutaneously to the dorsal surface of the scapulae. The catheters were flushed daily with 0.3 ml of sterile saline containing heparin (15 units) and penicillin B (60,000 units) to preserve catheter patency and prevent infection. After surgery, the rats were allowed to recover for 1 week prior to drug self-administration training. The catheters were flushed with 0.3 ml of heparinized saline (50 U/ml) every day, and if resistance or exudation was noted while flushing the catheters, we used 10 mg/kg propofol to assess the catheter patency. All catheters remained patent throughout the experiments.

### Self-administration

All the experiments were conducted in an operant chamber (AniLab Software Instruments Co., Ltd., Ningbo, China). After ~7 days of recovery from surgery, the rats (*n* = 16) were divided randomly into two groups, which underwent ketamine and 2F-DCK self-administration training. During the 4-h acquisition phase of self-administration starting with the green light inside the active nose-poke hole, rats were trained to respond under a fixed ratio 1 (FR1) reinforcement schedule for ketamine and 2F-DCK (0.5 mg·kg^−1^·infusion^−1^), in accordance with the protocol described in a previous study (Li et al., 2022). To prevent overdose, the number of infusions was limited to 200 per session. Each infusion was paired with a 20-s illumination of the house light in combination with the noise of the infusion pump. A time-out period was imposed for 20 s, during which the response produced no programmed consequences but was still recorded. Responding to inactive nose pokes produced no programmed consequences. Acquisition training under these conditions continued for at least 10 sessions until the animals met the stability criteria (±15% of the mean number of infusions of three consecutive training sessions).

### Behavioral economics demand curve

Behavioral economics demand was determined after stable self-administration training (Lai et al., 2022). Briefly, the dose (0.5 mg/kg/infusion) chosen was consistent with the training session, and each daily session was 4 h in duration. Once the number of infusions was stable under the FR1 schedule, the FR value was increased across sessions until zero infusions were delivered in a single session in the following order: 3, 10, 18, 32, 56, 98, 172, and 300 (i.e., each value was 1.75 times the preceding response requirement except in the case of 3 and 10). If the mean number of infusions for 2 consecutive days showed considerable variability at a particular ratio, three or more sessions were conducted, and the most discrepant result was excluded from the data analysis.

### Extinction and reinstatement

After the acquisition and determinations of self-administration for 14 days, rats (each group, *n* = 7) underwent an extinction period for seven sessions during which the drug was not available. Both cue- and drug-induced reinstatement tests were conducted for 2 h in operant chambers after extinction training. The cue-induced reinstatement test began with drug-associated conditioned cues (CS), consisting of the house light and the sound of the pump. Nose pokes in both the active and inactive holes were recorded. The only difference between the self-administration sessions and reinstatement tests was the lack of drug infusion. After the CS-induced relapse test was completed, the rats underwent another extinction training for three sessions, following which they were administered either ketamine (10 mg/kg, i.p.) or 2F-DCK (10 mg/kg, i.p.) and placed immediately into the operant chamber for the drug-induced reinstatement test without drug-associated cues. Nose pokes in both the active and inactive holes were recorded.

### Western blot

Another group of rats was used to investigate the expression of signaling proteins in the NAc. The groups of rats were divided into the control group (a drug-naive), the ketamine or 2F-DCK extinction group in which they completed 14 days of self-administration followed by 10 days of extinction, and the ketamine or 2F-DCK priming group in which rats were exposed to either acute ketamine (10 mg/kg, i.p.) or 2F-DCK (10 mg/kg, i.p.) injection after extinction with CS rewards. The rats were decapitated within 2 h after the extinction or relapse test. The brains were rapidly removed and dissected to obtain NAc tissue. Tissue samples obtained from individual rats were immediately homogenized on ice in ice-cold RIPA lysis buffer (20 mM Tris, pH 7.5; 150 mM NaCl; 1% Triton X-100; 2.5 mM sodium pyrophosphate; 1 mM EDTA; 1% Na_3_VO_4_; 0.5 mg/ml leupeptin; and 1 mM phenyl-methanesulfonyl fluoride) containing 1 mM phenyl-methanesulfonyl fluoride and 1 mM phosphatase inhibitor. After immersion for 30 min in RIPA lysis buffer, the homogenized tissue was centrifuged at 12,000 rpm at 4°C for 20 min. Protein concentrations were determined using a bicinchoninic acid protein assay, and the samples were further diluted in RIPA lysis buffer to equalize the protein concentrations. The extracts were then mixed with 5 × loading buffer, boiled for 5 min, centrifuged for 5 min, aliquoted, and stored at −80°C. For Western blot analysis, 20–40 μg of protein was subjected to electrophoresis on sodium dodecyl sulfate (SDS) polyacrylamide mini gels for 20 min at 100 V in the stacking gel and 60 min at 220 V in the resolving gel. After electrophoresis, the separated proteins were transferred to a polyvinylidene fluoride (PVDF) membrane at 100 V for 60 min. To detect the protein of interest, the membranes were blocked in 5% non-fat dried milk in Tris-buffered saline (TBS) for 3 h. Membranes were then probed with different primary antibodies. Antibodies for BDNF (1:1,000; Ab108319, Abcam), CREB (1:1,000; Ab32515, Abcam), p-CREB (1:500; 9198s, Cell Signaling Technology), ERK (1:500; 4695s, Cell Signaling Technology), p-ERK (1:500; 4370s, Cell Signaling Technology), Akt (1:1,000; 9272s, Cell Signaling Technology), p-Akt (1:1,000; 9271s, Cell Signaling Technology), GSK-3β (1:1,000; 12456-R, Cell Signaling Technology), p-GSK-3β (1:500; 5558-R, Cell Signaling Technology), mTOR (1:500; 2972s, Cell Signaling Technology), p-mTOR (1:500; 2971s, Cell Signaling Technology), or β-actin (1:2,000; 4967s, Cell Signaling Technology) were added to TBST (Tris-buffered saline plus 0.05% Tween-20, pH 7.4) containing 5% bovine serum albumin. The blots were incubated in the primary antibody solution overnight at 4°C. The next day, after three 10-min washes in TBST buffer, the membranes were incubated for 1 h at room temperature on a shaker with a 1:5,000 dilution of fluorescent secondary antibody [goat (polyclonal) anti-rabbit IgG (LI-COR Bioscience, Beijing, China)] in TBST in the dark. The blots were then washed three times for 10 min each in TBST and detected using the Odyssey Imaging System Application (Odyssey, USA).

### Data analyses

For acquisition studies on the FR1 schedule, results are shown as the mean ± standard error of the mean (SEM) of the number of responses and infusions across 10 sessions. Statistical tests were performed by using repeated measures analysis of variance (ANOVA). For the demand curve, as reported previously (Hursh and Silberberg, 2008), the logarithm of the number of infusions was plotted as a function of the logarithm of the price (FR value) according to the equation log *Q* = log *Q*_0_ + *k*(*e*^−α*QoC*^ −1), where Q is the average number of infusions obtained for a given drug at any FR value, Q_0_ is the average number of infusions obtained for a given drug when FR is set to 1, C is the FR value, α is the elasticity coefficient of each drug, and k is a constant. In this study, the k parameter was set to 1, and the log of the maximum number of infusions was obtained under any FR for a particular determination. Because the parameter α is inversely proportional to the reinforcing effectiveness, the equation converts it into an essential value (EV) with EV = 1/(100 α k^1.5^), which is directly related to the reinforcing effectiveness (Hursh and Silberberg, 2008; Maguire et al., 2020). For both normalized and non-normalized analyses, we also fitted the exponential equation to the individual subject data and obtained the best-fitting α and EV parameters for each rat. In cases where individual rats did not earn a reinforcer at a particular ratio, a value of 0.1 was assigned because the log of zero is undefined. For both CS- and drug-induced reinstatement tests, the results were expressed as mean ±SEM of the number of responses per session, and the analysis of differences between groups (ketamine *vs*. 2F-DCK) and tests (extinction *vs*. reinstatement) was performed by using a two-way ANOVA. Western blot data were analyzed using an independent *t*-test between the control and ketamine or 2F-DCK groups. Changes were considered statistically significant when P <0.05. Statistical analysis was performed using SPSS 18.0 (SPSS, Inc.) and GraphPad Prism 7 (GraphPad Software, Inc.).

## Results

### Comparison of reinforcing effectiveness between 2F-DCK and ketamine

**Figure 1:**
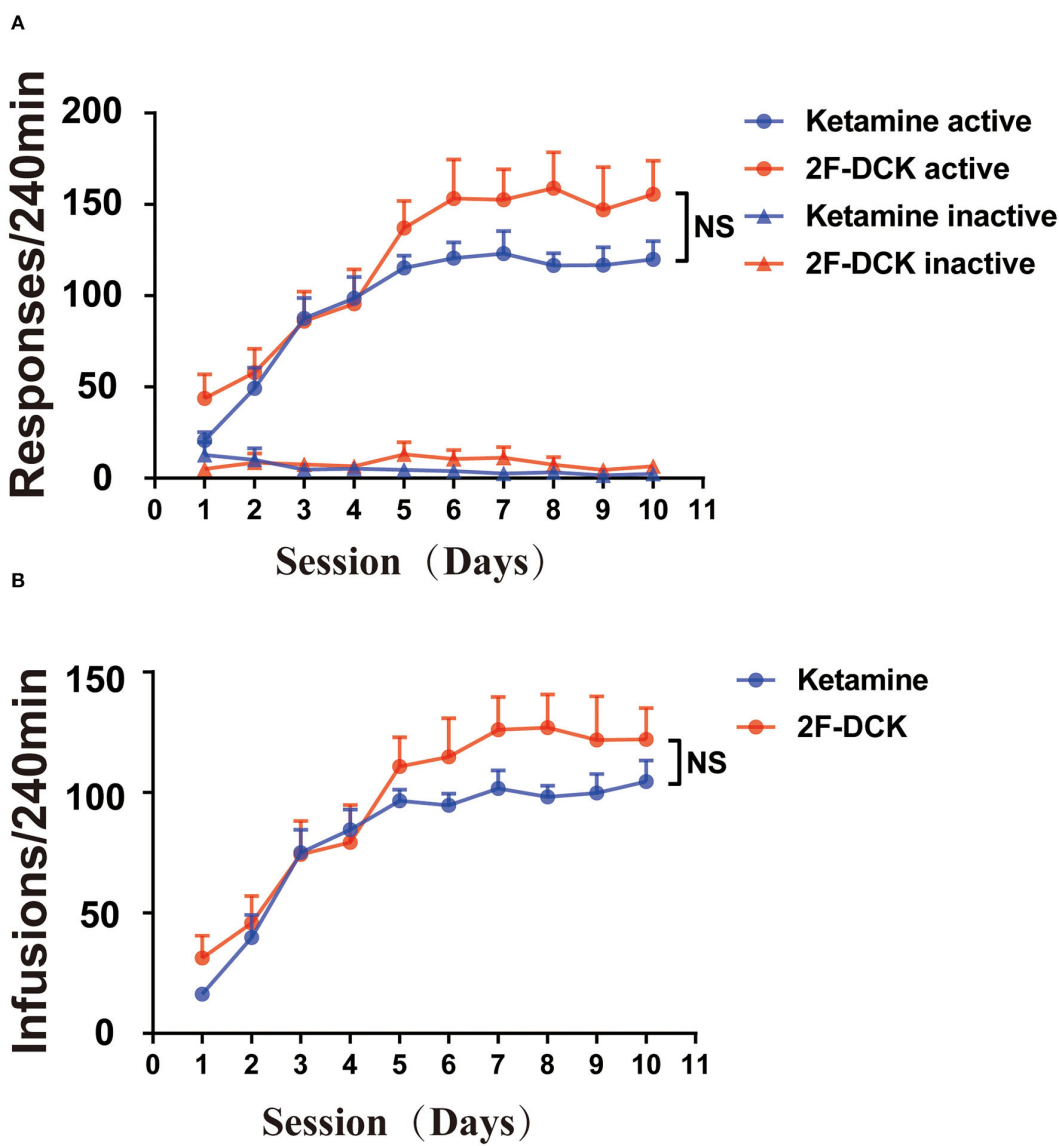
2F-DCK and ketamine self-administration. The rats were acquired and maintained at stable 2F-DCK or ketamine self-administration for 10 days. **(A)** The active or inactive response per 4 h of the training session was not different between 2F-DCK and ketamine groups under the FR1 schedule. **(B)** The total infusions per 4 h trained for 10 sessions were also not different between 2F-DCK and ketamine groups. Data are presented as mean ± SEM. NS, not significant.

Both 2F-DCK and ketamine quickly induced self-administration behavior, as shown in Figure 1. From the first day of training, the number of active responses increased significantly, and the number of injections increased significantly in the first 5 days. Moreover, the mean number of infusions on days 8, 9, and 10 met the stability criteria for acquisition of self-administration. Repeated-measures ANOVA revealed a significant increase in active responses in the 2F-DCK group with an increase in training days [*F*_(9,126)_ = 5.513, *P* < 0.001], along with a significant difference between the active and inactive responses [*F*_(1,14)_ = 45.74, *P* < 0.0001]. We also observed a significant increase in active responses [*F*_(9,126)_ = 4.828, *P* < 0.001] and between active and inactive responses [*F*_(1,14)_ = 167.6, *P* < 0.001]. No significant difference was observed between 2F-DCK and ketamine in active responses [*F*_(1,14)_ = 1.949, *P* = 0.184] and inactive responses [*F*_(1,14)_ = 0.128, *P* = 0.726]. The average number of daily injections in the last 3 days in the 2F-DCK and ketamine groups was 122.62 ± 7.93 and 101.58 ± 4.02, respectively, but the two groups showed no significant difference [*F*_(1,14)_ = 1.266, *P* = 0.280]. Thus, rats in both 2F-DCK and ketamine groups could quickly acquire and maintain self-administration, suggesting that 2F-DCK and ketamine have similar reinforcement and rewarding effects.

**Figure 2:**
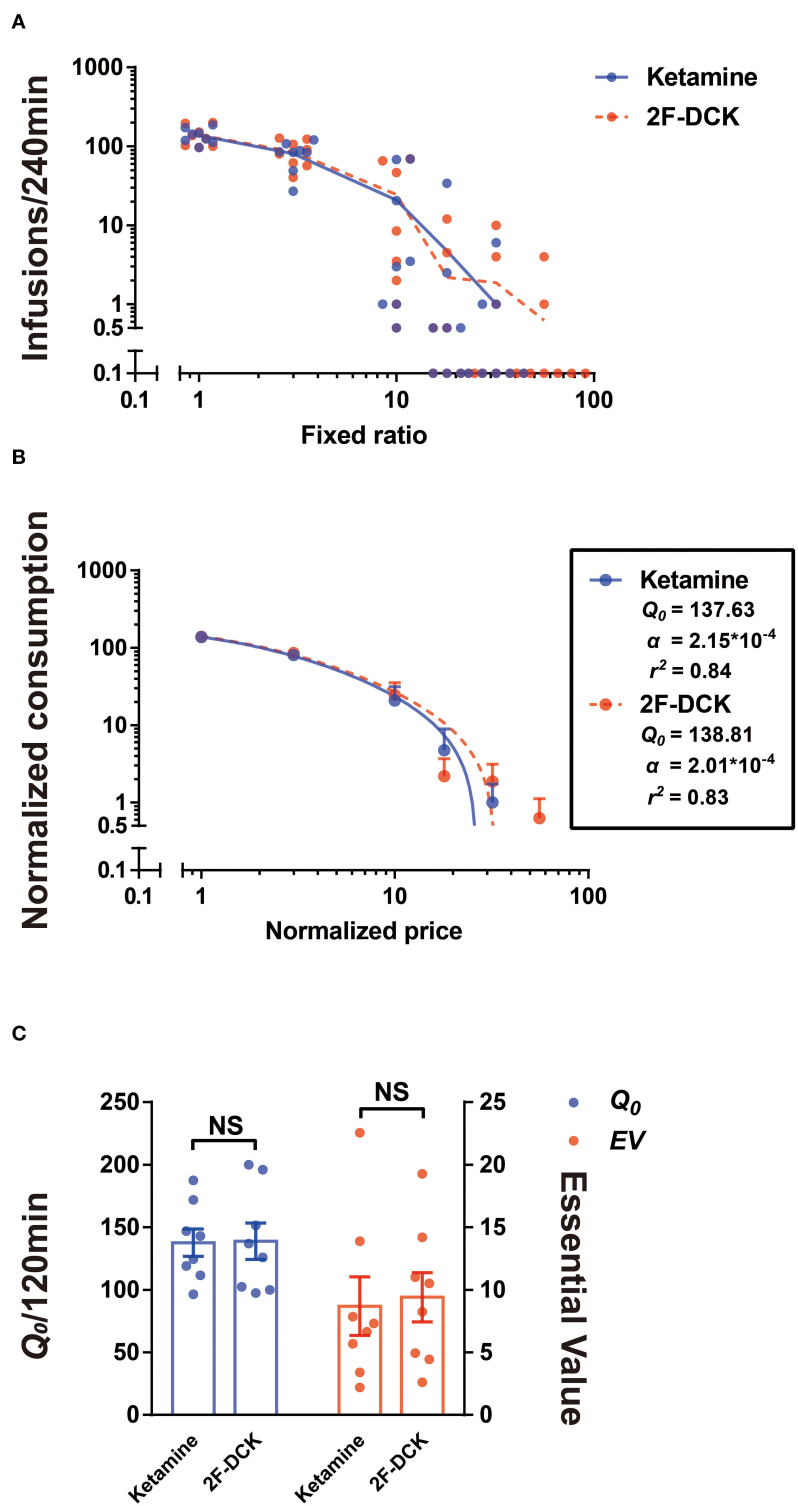
Demand curve for 2F-DCK and ketamine. **(A)** Individuals' demand curves for 2F-DCK and ketamine, in which the number of infusions is plotted as a function of the fixed-ratio requirement on a log scale. **(B)** Normalized demand curves for 2F-DCK and ketamine, and the abscissa and ordinate use logarithmic scaling. **(C)** Parameters derived from the demand curve fit for each of the solutions available for self-administration. Estimates of consumption at a minimal cost (Q_0_; Equation 1) are plotted in the left ordinate, whereas estimates of essential value (EV; Equation 2) are plotted in the right ordinate. NS, not significant.

In the actual test of economic demand, only FR10 was tested for 3 days and excluded the data on the second day for analysis due to considerable variability. To complete the test within the service life of the intravenous catheter, when the number of infusions of more than half of the rats is 0 and other rats gained ≤ 10 infusions, each FR value was only tested for 1 day. Figure 2 shows the non-normalized and normalized demand curves for ketamine and 2F-DCK. Subsequent fitting of the exponential equation to the normalized results from each rat individually revealed the α parameter in the ketamine group [2.145E-4 (95% CI: 1.918E-4, 2.365E-4)] and in the 2F-DCK group [2.014E-4 (95% CI: 1.638E-4, 2.588E-4)]. The α parameter was similar, and the 95% confidence interval almost coincided. As shown in Figure 2C, Q_0_ and EV values in the ketamine group were 137.63 ± 10.92 and 8.69 ± 2.33, respectively, and the Q_0_ and EV in the 2F-DCK group were 138.81 ± 14.56 and 9.40 ± 1.97, respectively. The independent *t*-test revealed no significant differences in Q_0_ and EV between the ketamine and 2F-DCK groups (*t* = 0.065, *P* = 0.949 and *t* = 0.234, *P* = 0.819). Together, these results showed that the elasticity coefficient and EV in the 2F-DCK and ketamine groups were very close, suggesting that 2F-DCK and ketamine functioned as reinforcers with similar effectiveness.

### Reinstatement of 2F-DCK and ketamine-seeking behaviors

**Figure 3:**
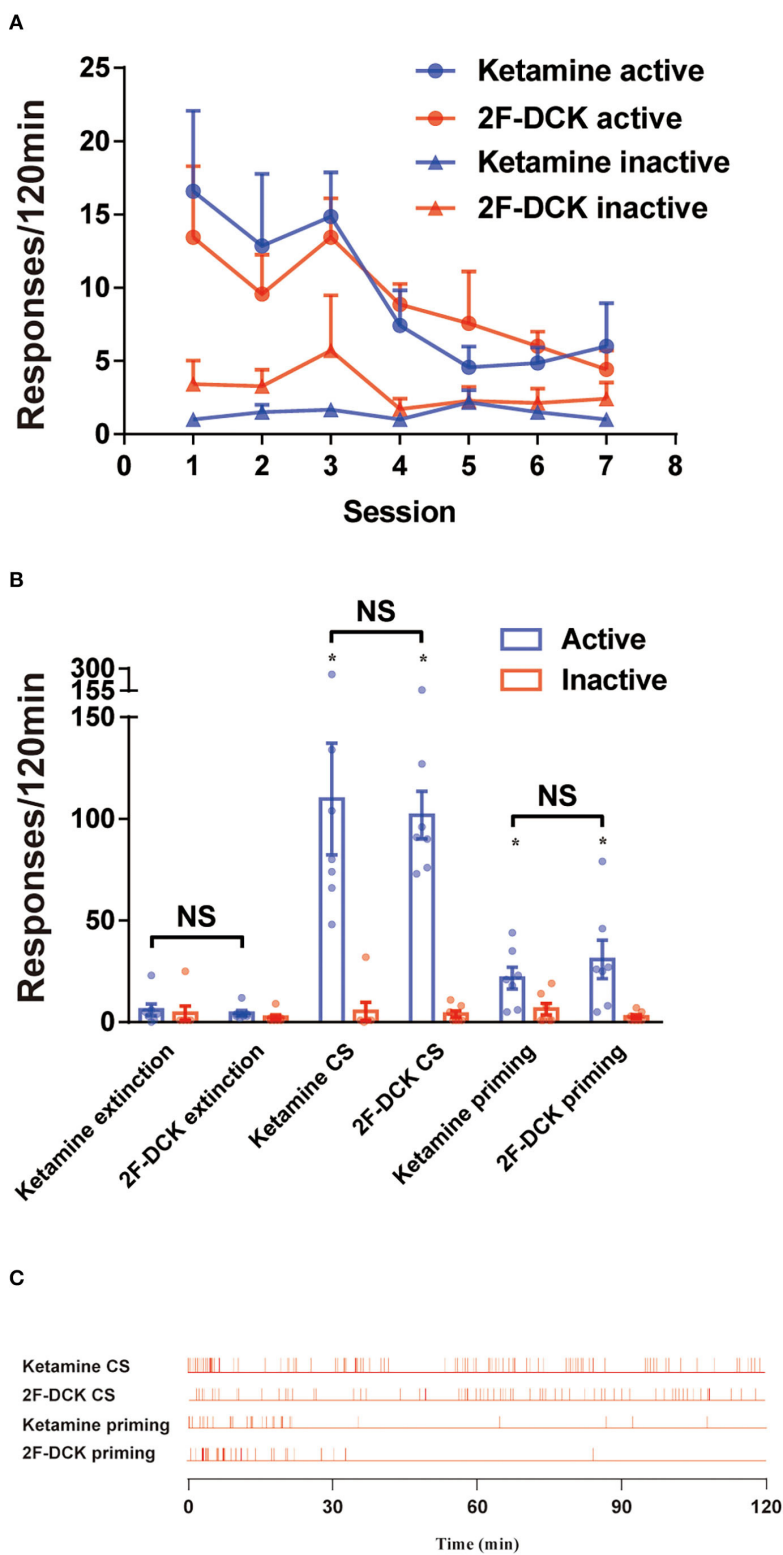
The extinction and reinstatement of 2F-DCK and ketamine. **(A)** The active or inactive responses between the 2F-DCK and ketamine groups per 2 h session for 7 days of extinction training were gradually decreased. **(B)** Mean (± SEM) number of responses during the final extinction session, cue-induced, and drug-priming reinstatement tests as a function of the group. There was a relative increase in responses from extinction to reinstatement in both the 2F-DCK and ketamine groups. Each bar represents mean ± SEM (*n* = 8 per group). *P < 0.05 in comparison with extinction. **(C)** The representative raster plot for active responses as a function of time during a 2-h reinstatement testing session. Ketamine- or 2F-DCK-associated CS-induced responses were completed across the session, but both ketamine and 2F-DCK priming responses were mostly completed within the first 30 min.

After stable self-administration training for 14 days, the rats completed the 2-h extinction period in self-administration chambers for 7 days. The results of extinction training is shown in Figure 3A. Two-way ANOVA was used to analyze the reinstatement of drug-seeking behavior induced by CS with extinction between 2F-DCK and ketamine-trained rats. As shown in Figure 3B, a significant difference was observed in the number of active responses between the extinction and CS-induced reinstatement (*F* = 44.797, *P* < 0.001); however, no significant difference was observed between 2F-DCK and ketamine groups (*F* = 0.098, *P* = 0.756) and interaction (*F* = 0.044, *P* = 0.836). Moreover, there were no differences in the inactive responses in tests (*F* = 0.167, *P* = 0.686), 2F-DCK and ketamine treated group (*F* = 0.306, *P* = 0.585), and interaction (*F* = 0.014, *P* = 0.905). These results suggest that both 2F-DCK and ketamine groups of rats showed significant drug-seeking behavior when exposed to CS which is associated with previous rewards. The two groups of rats were intraperitoneally injected with the same drugs as the drug-priming-induced reinstatement at doses of 10 mg/kg ketamine or 2F-DCK, and immediately placed into their own administration cages for 2 h. Two-way ANOVA analysis revealed a significant difference between extinction and reinstatement induced by priming (*F* = 13.662, *P* = 0.001), but no difference between 2F-DCK and ketamine groups (*F* = 0.441, *P* = 0.513) and interaction (*F* = 0.883, *P* = 0.357). Moreover, there was no difference in the inactive responses between the tests (*F* = 0.239, *P* = 0.629), 2F-DCK and ketamine treated group (*F* = 1.496, *P* = 0.233), and interaction (*F* = 0.135, *P* = 0.717). Figure 3C shows the frequency of active responses across 2-h reinstatement testing. These results suggest that both 2F-DCK and ketamine priming can produce an increase in drug-seeking behavior after extinction. The results demonstrated relapse and craving in the rats after exposure to the CS or 2F-DCK and ketamine lapse after extinction and withdrawal from 2F-DCK and ketamine self-administration.

### Relationship between reinforcing effectiveness and reinstatement

The relationship of the Q_0_ and α parameters with extinction, cue-induced, or drug-priming reinstatement behaviors is shown in Table 1. Spearman's rank-order correlation analysis showed that the Q_0_ parameter was not associated with the degree of extinction, cues, or drug-priming reinstatement. However, the α parameter was inversely related to the degree of reinstatement induced by cues or drug priming in the ketamine group and 2F-DCK group, but the statistical value in the 2F-DCK group was equal to 0.05.

**Table 1:** The relationship of the Q_0_ and α parameters with extinction, cue-induced, or drug-priming-reinstatement behaviors.

	**2F-DCK (*****n*** = **7)**			**Ketamine (*****n*** = **7)**		
	**Extinction**	**CS**	**Priming**	**Extinction**	**CS**	**Priming**
**Q**_**0**_	0.18 (0.70)	0.61 (0.15)	0.57 (0.18)	−0.58 (0.18)	−0.04 (0.94)	−0.04 (0.94)
**α**	−0.34 (0.45)	−0.75 (0.05)	−0.75 (0.05)	−0.45 (0.31)	−0.93 (0.003)^**^	−0.86 (0.014)^*^

Spearman's rank-order correlations revealed a significant negative correlation between α ranks and reinstatement ranks in the 2F-DCK and ketamine groups (

* P <0.05;

** p <0.01).

### Signaling protein expression in the NAc

**Figure 4:**
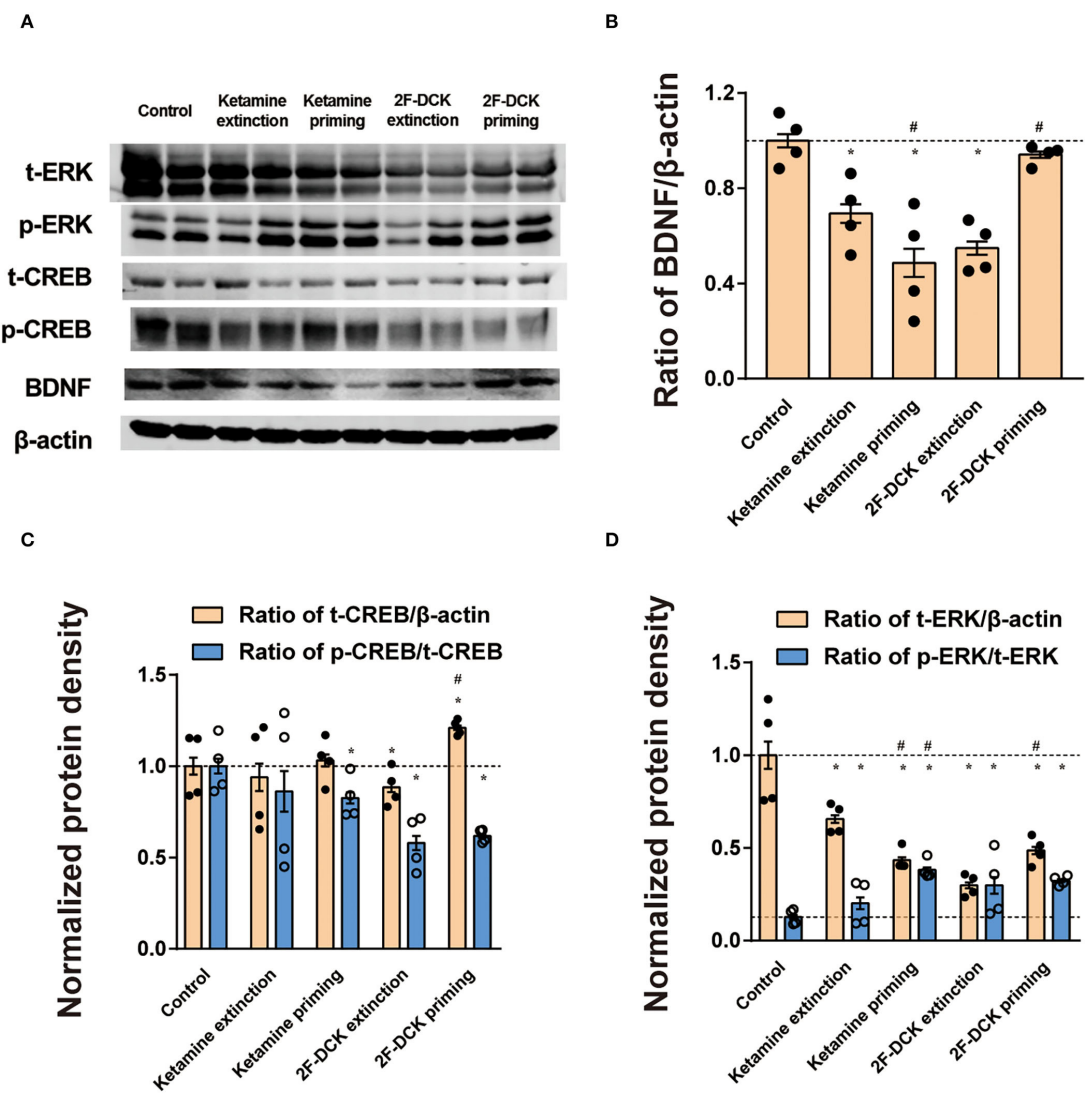
Expression of BDNF, CREB, ERK, CREB, and ERK phosphorylation in the NAc during extinction and reinstatement. **(A)** Representative bands of BDNF, CREB, and CREB phosphorylation and β-actin in the NAc, and quantification of Western blot bands, with each bar representing the mean ± SEM (*n* =4). **(B)** The histogram shows the relative expression of BDNF to β-actin among the groups. **(C)** The histogram shows the relative expression of t-CREB/β-actin and p-CREB/t-CREB. **(D)** The histogram is shown as the relative expression of t-ERK/β-actin and p-ERK/t-ERK. *P <0.05 in comparison with the control and ^#^*P* <0.05 in comparison with extinction.

The relative expression of BDNF, CREB, or ERK in the NAc is shown in Figure 4. The independent *t*-test showed a significant decrease in BDNF levels in ketamine extinction (*t* = 6.362, *P* < 0.01), 2F-DCK extinction (*t* = 11.507, *P* < 0.01), and ketamine priming group (*t* = 7.907, *P* < 0.01) compared with the control group, but no difference in the 2F-DCK priming group (*t* = 1.907, *P* = 0.075). Moreover, a decrease in the BDNF levels was observed in the ketamine priming group compared to that of the ketamine extinction group (*t* = 2.934, *P* < 0.01), but an increase in 2F-DCK priming rats was noticed compared to that in 2F-DCK extinction group (*t* = 12.773, *P* < 0.01). The total CREB expression was not different in the ketamine extinction (*t* = 0.689, *P* = 0.500) and priming groups (*t* = 0.561, *P* = 0.581), decreased in the 2F-DCK extinction group (*t* = 2.154, *P* = 0.045), and increased in the 2F-DCK priming rats (*t* = 4.407, *P* < 0.01), in comparison with the control group. Moreover, the p-CREB level decreased in 2F-DCK extinction (*t* = 7.564, *P* < 0.01), ketamine (*t* = 3.451, *P* < 0.01), and 2F-DCK (*t* = 9.316, *P* < 0.01) priming groups, but no difference in the ketamine extinction (*t* = 0.313, *P* = 0.759) group was noted when compared to the control. There was no difference either between ketamine extinction and priming groups (*t* = 0.313, *P* = 0.759) or between 2F-DCK extinction and priming groups (*t* = 0.946, *P* = 0.362). The total ERK levels were decreased significantly in four groups (ketamine extinction: *t* = 4.513; ketamine priming: *t* = 7.549; 2F-DCK extinction: *t* = 9.366; 2F-DCK priming: *t* = 6.777), and the relative expression of p-ERK increased significantly in four groups (ketamine extinction: t=2.220, P=0.044; ketamine priming: t=14.546, P <0.01; 2F-DCK extinction: *t* = 3.659, *P* <0.01; 2F-DCK priming: *t* = 15.174, *P* < 0.01) in comparison with the control group.

**Figure 5:**
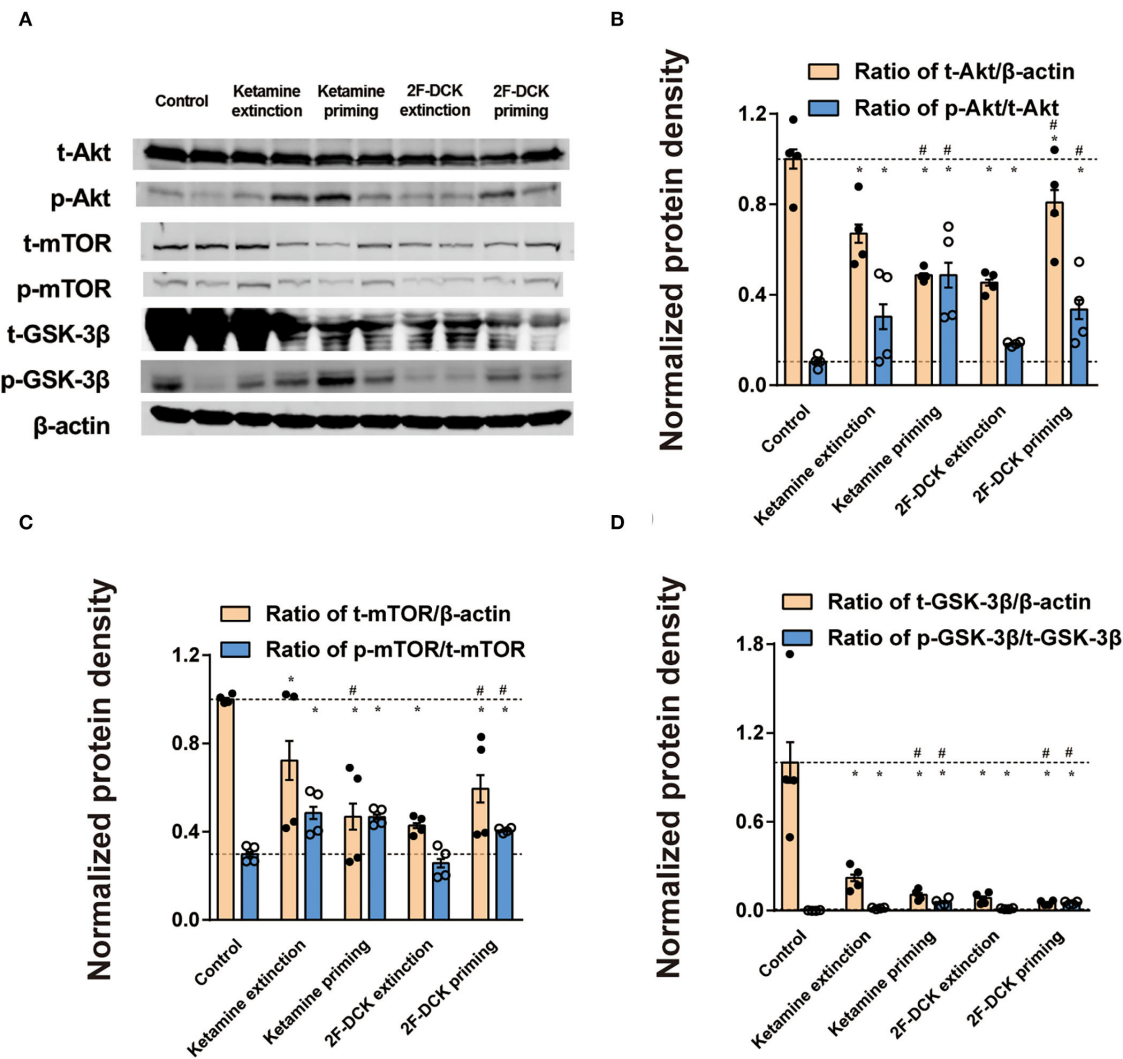
Expression of Akt, mTOR, and GSK-3β, and their phosphorylation in the NAc during the extinction and reinstatement. **(A)** Representative bands of Akt, mTOR, GSK-3β, and their phosphorylated forms, and quantification of Western blot bands (*n* = 4), with each bar representing the mean ± SEM. **(B)** The histogram shows the relative expression of t-Akt/β-actin and p-AKT/ t-Akt. **(C)** The histogram shows the relative expression of t-mTOR/β-actin and p-mTOR/ t-mTOR. **(D)** The histogram shows the relative expression of t-GSK-3β/β-actin and p-GSK-3β/t-GSK-3β. *P < 0.05 in comparison with control and ^#^*P* < 0.05 in comparison with extinction.

As shown in Figure 5, the relative expression levels of Akt, GSK-3β, and mTOR and their phosphorylation in the NAc were analyzed. There was a significant decrease in the t-Akt levels (ketamine extinction: *t* = 5.66, *P* < 0.01; ketamine priming: *t* = 11.917, *P* < 0.01; 2F-DCK extinction: *t* = 12.362, *P* < 0.01; 2F-DCK priming: *t* = 2.769, *P* = 0.011), t- GSK-3β levels (ketamine extinction: *t* = 5.645, *P* < 0.01; ketamine priming: *t* = 6.531, *P* < 0.01; 2F-DCK extinction: *t* = 6.702, *P* < 0.01; 2F-DCK priming: *t* = 6.940, *P* < 0.01), and t-mTOR levels (ketamine extinction: *t* = 3.124, *P* = 0.01; ketamine priming: *t* = 8.906, *P* < 0.01; 2F-DCK extinction: *t* = 44.482, *P* < 0.01; 2F-DCK priming: *t* = 6.498, *P* < 0.01) in comparison with those in the control; however, there was an increase in the p-Akt levels (ketamine extinction: *t* = 3.566, *P* < 0.01; ketamine priming: *t* = 6.854, *P* < 0.01; 2F-DCK extinction: *t* = 9.653, *P* < 0.01; 2F-DCK priming: *t* = 5.394, *P* < 0.01), p-GSK-3β levels (ketamine extinction: *t* = 9.912, *P* < 0.01; ketamine priming: *t* = 7.820, *P* < 0.01; 2F-DCK extinction: *t* = 10.472, *P* < 0.01; 2F-DCK priming: *t* = 17.581, *P* < 0.01), and p-mTOR expression (ketamine extinction: *t* = 6.406, *P* < 0.01; ketamine priming: *t* = 11.544, *P* < 0.01; 2F-DCK extinction: *t* = 1.914, *P* = 0.073; 2F-DCK priming: *t* = 9.939, *P* < 0.01) in comparison with those in the control. The expression levels of p-Akt, p-GSK-3β, and p-mTOR in the ketamine priming group (p-Akt: *t* = 2.352, *P* = 0.028; p-GSK-3β: *t* = 5.667, *P* < 0.01) or 2F-DCK priming group (p-Akt: *t* = 3.597, *P* < 0.01; p-GSK-3β: *t* = 13.092, *P* < 0.01; p-mTOR: *t* = 7.737, *P* < 0.01) were higher than those of ketamine extinction or 2F-DCK extinction groups, respectively, but no difference in the p-mTOR levels were noted between the ketamine extinction and priming groups (*t* = 0.607, *P* = 0.554).

## Discussion

The main findings of the present study are that both the α parameter and the EV of effectiveness between 2F-DCK and ketamine are similar, and the reinstatement of 2F-DCK- and ketamine-seeking behaviors after extinction is also similar. These data, along with the data from previous studies, suggest that 2F-DCK and ketamine have abuse potential and can cause cravings after extinction (Huang et al., 2015; Li et al., 2022]. The value of the α parameter is inversely proportional to the intensity of reinstatement of 2F-DCK- and ketamine-seeking behaviors, which could predict cravings for 2F-DCK and ketamine after withdrawal from self-administration. Interestingly, the expression of BDNF and phosphorylation of ERK, AKT, mTOR, GSK-3β, and CREB decreased in the NAc, while the phosphorylation of ERK, AKT, mTOR, and GSK-3β in the NAc increased after extinction and reinstatement of 2F-DCK and ketamine, suggesting that Akt, ERK, and CREB in the NAc may have different regulatory pathways, and downregulation of CREB/BDNF and upregulation of Akt/mTOR/GSK-3β may be involved in 2F-DCK and ketamine relapse and addiction after withdrawal.

The present study used a behavioral economics approach to compare the reinforcing efficacy of 2F-DCK and ketamine. With respect to drug self-administration, the elasticity of demand in animal self-administration has been suggested to partly correspond to abuse liability in humans (Hursh et al., 2005; Schwartz et al., 2019). The demand curves plot the amount of infusion consumed as a function of the price (response requirement) of 2F-DCK and ketamine. The change in the elasticity of the demand curve as the response increases reflects the reinforcing effectiveness (Hursh and Silberberg, 2008). The α parameter is a free parameter that reflects demand elasticity and is inversely related to the reinforcing effectiveness. It is transformed into an EV, which is directly related to reinforcing effectiveness (Hursh and Romaa, 2016; Zanettini et al., 2018; Maguire et al., 2020). Our previous studies have shown that 2F-DCK produces self-administration, conditions place preference, and generalizes discriminative stimuli effects similar to ketamine (Li et al., 2022). Behavioral economics provides a framework for quantifying 2F-DCK and ketamine abuse potential, while also offering an approach complementary to the self-administration and dose–response curve used previously to characterize the abuse-related effects of 2F-DCK and ketamine (Li et al., 2022). Thus, the α parameter and EV values of 2F-DCK and ketamine were very close, indicating that both have similar reinforcing effectiveness.

Environmental cues associated with rewards can acquire motivational properties (Kuijer et al., 2020). The present results showed that repeated exposure to context could extinguish the ketamine- or 3F-DCK-seeking behavior in their chambers after abstinence, supporting the idea that treatments for ketamine use disorders could be improved by considering drug-associated contexts as a factor in extinction interventions (Kuijer et al., 2020). Several factors, such as the presentation of either drug-associated cues or a single dose of the drug itself, are known to contribute to craving and relapse to drug use in the reinstatement model (Ma et al., 2019). The rats exposed to the CS could induce the elevation of 2F-DCK- or ketamine-seeking responses across a 2-h session after extinction in the present study. When the CS was paired previously with 2F-DCK or ketamine reward, it acquired the incentive properties and its ability to reinstate reward-seeking behavior (Perry et al., 2014). While in the rats exposed to non-contingent 2F-DCK or ketamine injection, the responses were completed mostly within the first 30 min. The intensity and duration of drug-seeking behavior induced by CS are obviously greater than that induced by non-contingent 2F-DCK and ketamine injections, suggesting that both 2F-DCK and ketamine could produce drug cravings or relapse on exposure to drug-associated CS or the drug itself after abstinence. To our knowledge, this is the first study to show a relationship between the economic demand parameter α and the reinstatement of drug seeking. For the self-administered 2F-DCK and ketamine groups, the α parameter was inversely related to the degree of CS-induced or drug-priming reinstatement. These results are consistent with the relationship between the methamphetamine demand curve and relapse (Galuska et al., 2011). Demand elasticity not only provides a metric to rank-order ketamine or 2F-DCK of abuse in terms of abuse liability but may also predict the propensity to ketamine or 2F-DCK relapse.

The CREB is an important transcription factor involved in the regulation of many processes, including synaptic plasticity, memory, and addiction (Nestler, 2004). The CREB family transcription factors are the main regulators of BDNF gene expression after tropomyosin receptor kinase B (TrkB) signaling (Esvald et al., 2020). The present results showed a decrease in the p-CREB/CREB ratio and the BDNF level in the NAc under CS and priming conditions. This result is different from the increased expression of BDNF following a single injection of ketamine (Li et al., 2010; Pham and Gardier, 2019), which may be attributable to long-term exposure and withdrawal from ketamine or 2F-DCK. In a ketamine-induced schizophrenia-like deficit model, ketamine also reduced BDNF levels. These data were further supported by the findings obtained with the PDE1 selective inhibitor vinpocetine, which elevated BDNF expression (Ahmed et al., 2018). Ketamine is proposed to enhance BDNF release and subsequently promote protein synthesis in the mesolimbic brain regions known to regulate natural and drug rewards (Zanos and Gould, 2018). In the present study, the total protein expression of Akt, GSK-3β, mTOR, and ERK decreased significantly in the NAc and decreased in the NAc parallel to BDNF. BDNF is a CREB-dependent gene that plays a pivotal role in drug addiction (Sun et al., 2015). Neutralizing endogenous BDNF regulation with intra-NAc infusions of antibodies to BDNF subsequently reduced cocaine self-administration and attenuated relapse. Dynamic induction and release of BDNF from neurons in the NAc during cocaine use promote the development and persistence of addictive behavior (Graham et al., 2007). Endogenous BDNF acts on the TrkB receptor to provide an inhibitory tone for reinstated cocaine-seeking, and this effect was recapitulated by exogenous BDNF (Bobadilla et al., 2019). It is reasonable to speculate that downregulation of CREB-BDNF signaling could account for 2F-DCK and ketamine relapse.

Classically, dopamine receptors have been shown to regulate cAMP/PKA and Ca^2+^ pathways through G protein-mediated signaling. A major downstream target of striatal D1R is the ERK pathway. ERK activation by drugs of abuse acts as a key integrator of D1R and glutamate NMDA receptor (NMDAR) signaling (Cahill et al., 2014). NMDARs have been linked with ERK activation in the NAc (Girault et al., 2007). Activation of the NMDAR-D1R/ERK/CREB signal transduction pathway plays a critical role in the control of reward-seeking behavior through reward-predictive cues (Kirschmann et al., 2014). In conjunction with studies showing increased striatal CREB phosphorylation in response to the application of NMDA or activation of D1Rs (Kirschmann et al., 2014), decreased CREB phosphorylation in the NAc may account for the blockage of NMDA receptors by 2F-DCK and ketamine priming. A decrease in phosphorylated CREB and ERK in the NAc is associated with heroin seeking induced by cues after withdrawal (Sun et al., 2015). D1 receptor and ERK/CREB signaling in the ventral hippocampus and medial prefrontal cortex is associated with the formation of opiate-related associative memories (Rosen et al., 2016; Wang et al., 2019). In contrast, an increase in phosphorylated ERK expression in the NAc during 2F-DCK or ketamine relapse was observed. Thus, the molecular mechanism underlying 2F-DCK or ketamine relapse warrants further study.

Dopamine receptor D2 functions through the Akt/GSK-3β signaling cascade (Rosen et al., 2016; Wang et al., 2019). The present results showed upregulation of the Akt/mTOR/GSK-3β pathway, which is consistent with the previous reports (Zhu et al., 2021). Ketamine self-administration decreased the expression of GSK-3β in the NAc (Huang et al., 2015). Silencing of GSK-3β in NAc increases depression- and addiction-related behavior (Crofton et al., 2017). Ketamine at a lower dose induces behavioral sensitization, accompanied by an increase in spine density in the NAc and changes in protein expression in pathways commonly implicated in addiction (Strong et al., 2017). Three weeks of abstinence from ketamine was associated with increased mushroom spines in all the groups (Strong et al., 2019). The upregulation of AKT/mTOR signaling is associated with rapid-acting antidepressant-like effects. This requires AMPA receptor and mTOR activation (Lu et al., 2015) *via* BDNF and protein neo-synthesis (Pham and Gardier, 2019). Depression and addiction may share overlapping neural circuitry and molecular mechanisms; however, there is much that remains to be known about the neurobiological mechanisms underlying ketamine addiction (Kokane et al., 2020; Sial et al., 2020). Evidence has shown that the CREB/BDNF or Akt/GSK-3β signaling pathways may play critical roles in methamphetamine-induced neurotoxicity (Keshavarzi et al., 2019). The Akt/GSK-3β/mTOR signaling pathway is involved in the antidepressant-like effect of atorvastatin in mice (Ludka et al., 2016). According to the obtained data, the reinstatement of ketamine or 2F-DCK could probably be produced by the mediation of the CREB/BDNF or Akt/mTOR/GSK-3β signaling pathways.

In conclusion, the results demonstrated that 2F-DCK has similar reinforcing effectiveness and craving as ketamine after abstinence and suggested that the downregulation of CREB/BDNF and the upregulation of phosphorylation of Akt/mTOR/GSK-3β signaling pathway in the NAc may be involved in ketamine or 2F-DCK relapse.

## Data availability statement

The raw data supporting the conclusions of this article will be made available by the authors, without undue reservation.

## Ethics statement

The animal study was reviewed and approved by the Ethics Committee of Ningbo University.

## Author contributions

HD, ML, DZ, and DF performed research, analysis of the data, and the writing of the paper. YZ, SC, FW, and ZX performed research. HL, YW, and PX were responsible for the maintenance of animal and experimental conditions. WZ was responsible for the study design and revising the paper. All authors contributed to the article and approved the submitted version.

## Funding

This work was supported by the National Natural Science Foundation of China (82071499 and 81671321), Ningbo Public Welfare Research Project (202002N3169), the Open Project of Key Laboratory of Drug Monitoring and Control, Ministry of Public Security (2021-KLDMC-03), and the Zhejiang Medical and Health Leading Academic Discipline Project (00-F06).

## Conflict of interest

The authors declare that the research was conducted in the absence of any commercial or financial relationships that could be construed as a potential conflict of interest.

## Publisher's note

All claims expressed in this article are solely those of the authors and do not necessarily represent those of their affiliated organizations, or those of the publisher, the editors and the reviewers. Any product that may be evaluated in this article, or claim that may be made by its manufacturer, is not guaranteed or endorsed by the publisher.
